# Tempol, an Intracellular Antioxidant, Inhibits Tissue Factor Expression, Attenuates Dendritic Cell Function, and Is Partially Protective in a Murine Model of Cerebral Malaria

**DOI:** 10.1371/journal.pone.0087140

**Published:** 2014-02-28

**Authors:** Ivo M. B. Francischetti, Emile Gordon, Bruna Bizzarro, Nidhi Gera, Bruno B. Andrade, Fabiano Oliveira, Dongying Ma, Teresa C. F. Assumpção, José M. C. Ribeiro, Mirna Pena, Chen-Feng Qi, Ababacar Diouf, Samuel E. Moretz, Carole A. Long, Hans C. Ackerman, Susan K. Pierce, Anderson Sá-Nunes, Michael Waisberg

**Affiliations:** 1 Laboratory of Malaria and Vector Research, National Institute of Allergy and Infectious Diseases, National Institutes of Health, Rockville, Maryland, United States of America; 2 Laboratory of Immunogenetics, National Institute of Allergy and Infectious Diseases, National Institutes of Health, Rockville, Maryland, United States of America; 3 Laboratory of Experimental Immunology, Department of Immunology, Institute of Biomedical Sciences, University of São Paulo, São Paulo, SP, Brazil; 4 Laboratory of Parasitic Diseases, NIAID/NIH, Bethesda, Maryland, United States of America; 5 University of Virginia, Department of Pathology, Charlottesville, Virginia, United States of America; Federal University of São Paulo, Brazil

## Abstract

**Background:**

The role of intracellular radical oxygen species (ROS) in pathogenesis of cerebral malaria (CM) remains incompletely understood.

**Methods and Findings:**

We undertook testing Tempol—a superoxide dismutase (SOD) mimetic and pleiotropic intracellular antioxidant—in cells relevant to malaria pathogenesis in the context of coagulation and inflammation. Tempol was also tested in a murine model of CM induced by *Plasmodium berghei* Anka infection. Tempol was found to prevent transcription and functional expression of procoagulant tissue factor in endothelial cells (ECs) stimulated by lipopolysaccharide (LPS). This effect was accompanied by inhibition of IL-6, IL-8, and monocyte chemoattractant protein (MCP-1) production. Tempol also attenuated platelet aggregation and human promyelocytic leukemia HL60 cells oxidative burst. In dendritic cells, Tempol inhibited LPS-induced production of TNF-α, IL-6, and IL-12p70, downregulated expression of co-stimulatory molecules, and prevented antigen-dependent lymphocyte proliferation. Notably, Tempol (20 mg/kg) partially increased the survival of mice with CM. Mechanistically, treated mice had lowered plasma levels of MCP-1, suggesting that Tempol downmodulates EC function and vascular inflammation. Tempol also diminished blood brain barrier permeability associated with CM when started at day 4 post infection but not at day 1, suggesting that ROS production is tightly regulated. Other antioxidants—such as α-phenyl N-tertiary-butyl nitrone (PBN; a spin trap), MnTe-2-PyP and MnTBAP (Mn-phorphyrin), Mitoquinone (MitoQ) and Mitotempo (mitochondrial antioxidants), M30 (an iron chelator), and epigallocatechin gallate (EGCG; polyphenol from green tea) did not improve survival. By contrast, these compounds (except PBN) inhibited *Plasmodium falciparum* growth in culture with different IC_50s_. Knockout mice for SOD1 or phagocyte nicotinamide adenine dinucleotide phosphate (NADPH) oxidase (gp91^phox–/–^) or mice treated with inhibitors of SOD (diethyldithiocarbamate) or NADPH oxidase (diphenyleneiodonium) did not show protection or exacerbation for CM.

**Conclusion:**

Results with Tempol suggest that intracellular ROS contribute, in part, to CM pathogenesis. Therapeutic targeting of intracellular ROS in CM is discussed.

## Introduction

Cerebral malaria (CM), caused by *Plasmodium falciparum*, is the deadliest form of malaria and is responsible for the deaths of approximately 500,000 humans each year, particularly children in sub-Saharan countries. Several pathogenic mechanisms reportedly contribute to morbidity and mortality in malaria, including parasite-related events such as sequestration and host response to infection, as recently reviewed by Miller et al. [1]. In malaria— and other infectious diseases—this response is illustrated by endothelial cell (EC) activation, leukocyte adhesion, impaired microcirculation, metabolic changes, apoptosis, and exacerbated inflammatory response leading to impairment of neurologic functions and organ dysfunction [2]–[9]. More recently, tissue factor (TF) has been identified in the endothelium of children who died from CM. In addition, parasitized red blood cells (pRBC) were found to support formation of multimolecular coagulation complexes such as prothrombinase and intrinsic Xnase [10]. The biochemical and cellular events triggered by activation of the coagulation cascade in CM have been explained by the “tissue factor model” [4], [11] This model places TF as the link between coagulation activation, inflammation, EC activation, and sequestration observed in CM. The TF model also emphasizes amplification of the coagulation cascade by pRBC and platelets—particularly at sequestration sites—as critical components of the disease [4], [11]. This leads to generation of thrombin, resulting in proteinase-activated receptor (PAR) activation and inflammation [12]. In the brain, coagulation reactions are exacerbated due to low levels of thrombomodulin, the cofactor for protein C activation [13]-[16]. The magnitude of pro- and anticoagulant responses results in a compensated or decompensated disseminated intravascular coagulation in malaria, as reviewed elsewhere [9], [11]. Hemostatic dysregulation in malaria has also been associated with lower nitric oxide (NO) bioavailability, complement activation, platelet GPIb shedding, microparticle formation, release of prohemostatic parasite molecules, and formation of ultralarge von Willebrand factor aggregates [10]–[13], [17]–[25]. Altogether, these results highlight participation of several components of vascular biology in malaria pathogenesis [6] and as targets for adjuvant therapy [26]–[29]. Furthermore, derangement of the coagulation is not specific for humans, as it takes place in monkeys [30]–[32], mice [12], [33], [34], and birds [35] infected with *Plasmodium* spp.

Inflammation is associated with an increase in oxidative stress, and involvement of reactive oxygen species (ROS) in human or experimental malaria has been consistently documented [36], [37]. Several mechanisms account for increased ROS in *P. falciparum* infection. Host response to *P. falciparum* infection activates cells that play a definitive role in immune and vascular inflammation [9], [38]. For example, *P. falciparum* merozoites and soluble antigens activate neutrophil and monocytes, resulting in production of ROS in vitro. *Pf*-GPI released during schizogony is a potent Toll-like receptor (TLR) agonist, inducing macrophage and dendritic cell (DC) activation [39]. Moreover, replication inside RBCs leads to hemolysis and therefore to production of cell-free hemoglobin which—once exposed to ROS—is oxidized and releases its heme prosthetic groups. These reactions produce intense oxidative burst through Fe^2+^ and Fenton reactions and may also result in apoptosis [40]. Further, metabolic changes such as restriction of the bioavailability of serum iron to *Plasmodium* have been described as a mechanism of disease control but may result in Fe^2+^ overload in tissues that can be cytotoxic, promoting tissue damage and exacerbating disease severity [41]–[43]. It has also been described that granulocytes obtained from children with severe malaria exhibit increased production of ROS compared with matched controls [44], [45]. Finally, malondialdehyde plasma levels (a marker of lipid oxidation) [46] or urinary F2-isoprostane (marker of oxidative stress) [47] are increased in malaria patients, while antioxidant levels (e.g. ascorbate, α-tocopherol, catalase) are suppressed [37], [48]–[50]. These results indicate that unbalanced production of free radicals takes place in the disease and also underscores the systemic component of *P. falciparum* infection, which is certainly not restricted to the brain.

ROS are generated extracellularly or intracellularly, either through activation of nicotinamide adenine dinucleotide phosphate (NADPH) oxidase (e.g. NOX2)—which is particularly abundant in phagocytes [51], or generated in the mitochondria [52], [53]. Importantly, cellular stressors (e.g. low oxygen, thrombin, oxidized LDL, glucose, angiotensin II, ROS) increase intracellular mitochondrial ROS production, which plays a major role in promoting endothelial dysregulation via activation of ROS-sensitive intracellular signaling pathways and redox-sensitive kinases (e.g. ASK1, MAPKs, PI3K, PTEN, mTOR, protein tyrosine phosphatases) and transcription factors (e.g. NF-κB, AP-1, and Egr-1) [52]–[56]. Therefore, intracellular ROS are considered signaling molecules. Because of their reactive nature, ROS also causes macromolecular damage of lipids, proteins, and DNA, which can lead to cell death. Further, superoxide (O_2_^−^) reacts with nitric oxide (NO) and as such reduces NO bioavailability and anti-inflammatory functions [52]–[56]. These events result in vasoconstriction, loss of anti-inflammatory and anti-adhesive function of NO, and activation of NF-κB, which promotes TF expression on one hand and induces expression of VCAM-1, selectins, monocyte chemoattractant protein (MCP-1), IL-6, and IL-8 on the other. Notably, increase for these markers of inflammation has been reported in CM [1]–[9].

Due to its role in inflammation, therapeutic targeting of intracellular antioxidants has been tested as an approach to reduce inflammation [57], [58]. A trial with 100 patients did not demonstrate a protective effect of N-acetylcysteine (NAC) when given together with antimalarial agents for CM [47]. Likewise, trials with desferoxamine in the treatment of pediatric CM have not shown consistent results [59]. In mice, administration of a soluble derivative of vitamin E (Trolox) or a combination of PEG-catalase and PEG- superoxide dismutase (SOD) partially increased survival [60]; however, others have neither found evidence for a role of ROS in experimental CM (ECM) [61] nor reported higher levels of ROS or reactive nitrogen species in the brain stem or cerebellum, or yet, total protein carbonylation (a marker of oxidative stress) [62]. More recently, it has been found that desferoxamine and NAC did not prevent mice from developing CM or cognitive dysfunction unless given with antimalarial agents [63], [64].One possible explanation for the lack of effects of these antioxidants in changing the survival curve for ECM—or as therapeutic agents in human malaria—is the fact that intracellular ROS remain relatively inaccessible to direct or indirect effects of some of these drugs [65], resulting in sustained inflammation.

Among several antioxidants currently available, Tempol is a redox-cycling (catalytic), metal-independent, and membrane-permeable antioxidant [57], [58]. It is a particularly attractive molecule because it promotes metabolism of O_2_^−^ at rates that are similar to SOD and is therefore considered a SOD mimetic; however, Tempol also facilitates metabolism of a wide range of ROS and reactive nitrogen species, including hydroxyl radicals (OH^−^), and exhibits catalase activity that further prevents generation of OH^−^ and H_2_O_2_ by Fenton reactions. Accordingly, Tempol is considered a general-purpose redox-cycling agent rather than a specific SOD mimetic. Tempol improves NO bioavailability and catalytically removes the highly reactive peroxynitrite (ONOO^−^) species that is produced by the reaction between O_2_^−^ and NO. Tempol has also been studied in several models of oxidative stress [57], [58]. Of relevance to malaria, Tempol was found to protect many organs—including the brain and the heart—from ischemia/reperfusion injury and improved survival in several models of shock. It also reduces brain or spinal cord damage after ischemia or trauma, among several other effects. In addition, Tempol attenuates the cerebral levels of malondialdehyde and the hippocampal levels of myeloperoxidase caused by cerebral ischemia and reperfusion. Tempol also interferes with metabolism, such as improving insulin response in diabetes, reducing weight gain, lowering blood pressure, and increasing the life span of mice [57], [58]. More recently, Tempol was found to decrease radiation damage and, for this reason, has been used in humans as a topical agent to prevent radiation-induced alopecia [66].

Here, we report on the use of Tempol in several in vitro assays using cell types (ECs, platelet DCs, and neutrophils) that play a definite role in malaria pathogenesis. We also evaluated the effects of Tempol in a murine model of CM and complemented our results using KO mice for phagocyte NADPH oxidase (Gp91^phox–/–^) and for SOD1. Pharmacological inhibition of SOD and NADPH oxidase has also been evaluated. Our results indicate that intracellular ROS play a partial role in CM pathogenesis, making them a potential therapeutic target in *P. falciparum* infection.

## Materials and Methods

### Materials

Tempol (4-hydroxy-Tempo; 4-hydroxy-2,2,6,6-tetramethylpiperidine-N-oxyl, 176141), epigallocatechin ((-)-epigallocatechin gallate [EGCG], E4143), M30 (dihydrochloride([5-(N-methyl-N-propargyaminomethyl)-8-hydroxyquinoline], SML0128), diethyldithiocarbamate (DETC), diphenyleneiodonium (DPI) and resazurin sodium were from Sigma-Aldrich (St. Louis, MO, USA). PBN (α-phenyl N-tertiary-butyl nitrone; 203995) was from Calbiochem (San Diego, CA, USA). MnTBAP (Mn(III)tetrakis(4-benzoic acid)porphyrin chloride) and Mitotempo (2,2,6,6-tetramethyl-4-[5-(triphenylphosphonio)pentoxy]piperidin-1-oxy bromide) were from Santa Cruz Biotechnology (Santa Cruz, CA, USA), Mitoquinone (MitoQ) was from BioTrend Chemicals (Destin, FL, USA). Human FX was from Hematologic Technologies (Essex Junction, VT, USA) or Enzyme Research Laboratories (South Bend, IN, USA). Recombinant human FVIIa (NovoSeven) was from Novo Nordisk (Plainsboro, NJ, USA). Chromogenic substrate S-2222 was purchased from Diapharma Group Inc. (Westchester, OH, USA). Ultrapure lipopolysaccharide (LPS-EB; TLR4 ligand) was from Invivogen (San Diego, CA, USA). Enzyme-linked immunosorbent assay (ELISA) for TF (Immunobind TF) was from American Diagnostica/Sekisui Diagnostics, LLC (Stamford, CT, USA). Horm fibrillar collagen and Luciferin-luciferase reagent were from Chrono-Log Corp. (Haverstown, PA, USA). ELISA for human IL-6, IL-8, and MCP-1 were from R&D Systems (St. Paul, MN, USA). OptEIA ELISA sets for murine TNF-α, IL-6, IL-12p70, IL-2, IFN-γ, and flow cytometry antibodies for CD11c, CD40, CD80, CD86, and MHC class II (I-A/I-E) cell surface markers were from BD Biosciences (San José, CA, USA).

### Culture of Human Dermal Microvascular ECs (MVECs)

Adult pooled MVECs were purchased from Lonza (Walkersville, MD, USA) and grown at 37°C, 5% CO_2_, in T-25 flasks in the presence of EBM-2 Plus (EBM-2 medium containing 2% fetal bovine serum and Single Quotes [human fibroblast growth factor, vascular endothelial growth factor, R3-insulin-like growth factor, ascorbic acid, human epidermal growth factor, and gentamicin-amphotericin B]) as described [26]. After trypsinization, MVECs were seeded at a density of 3×10^4^ cells/cm^2^ in flat-bottom tissue culture-treated 96-well plates (Costar 3596), and grown until confluence.

### Assembly of the Extrinsic Xnase by MVECs to Estimate TF Expression

MVECs (grown in 200 µL EBM-2 Plus) were incubated overnight with 0, 1, and 3 mM Tempol (diluted in EBM-2 Plus). Wells were then washed and replaced with 200 µL of 200 ng/mL of ultrapure LPS (in EBM-2 Plus). Alternatively, 200 µL of TNF-α (20 ng/mL in EBM-2 only, without FBS or Single Quotes) was added to the wells. Negative controls did not contain LPS or TNF-α. After six hours, wells were washed three times with 200 µL phenol red-free EBM-2 medium containing 0.3% BSA (no FBS or Single Quotes were added). Then, a mixture of 200 µL FX (50 nM) and FVIIa (5 nM) in EBM-2-BSA 0.3% (no FBS or Single Quotes) was added. After overnight incubation at 37°C, 5% CO_2_, 95 µL was removed, placed in another plate, and 5 µL S2222 (250 µM, final concentration) was added to start reactions. Hydrolysis was detected using a VersaMax ELISA microplate reader (Molecular Devices, Sunnyvale, CA, USA) equipped with a microplate mixer and heating system as described [26]. Reactions were continuously recorded at 405 nm for one hour at 37°C. FXa concentration was estimated using a standard curve performed under identical conditions.

### ELISA for TF

Wells of 96-well plates containing confluent MVEC were incubated overnight with Tempol in octoplicates followed by addition of LPS (200 ng/mL) or TNF-α (20 ng/mL) exactly as described above. Wells were washed twice with EBM-2 only (no FBS, Single Quotes, or BSA). To each well, 50 µL of TBS containing Triton X-100 (0.1%, v/v) was added. After 30 minutes at room temperature (RT), the plates were frozen at −80°C and two cycles of freeze-and-thaw were carried out. Then, 50 µL from four wells were combined in one Eppendorf and 50 µL of the other four wells were combined in another Eppendorf. This allowed the measurement to be performed in duplicate for each condition. Eppendorfs were centrifuged for ten minutes at 14,000×*g* in a bench centrifuge at RT. One hundred µL of each supernatant was used (without dilutions) to estimate human TF antigen with Immunobind tissue factor ELISA kit (American Diagnostica) as described [26]. A standard curve was carried out simultaneously in the same plate.

### Real-Time PCR for TF

T-25 flasks were seeded with MVECs that were allowed to grow until near confluency. Tempol (in EBM2-Plus) was then added at 0, 1, and 3 mM and incubated overnight. Flasks were washed twice with EBM-2 (no additions), and LPS (200 ng/mL in EBM-2 Plus) was added to the adherent cells. After 120 minutes, cells were washed with EBM-2 (no additions) and trypsinized as above. The pellet was subsequently used for extraction of RNA. Total RNA was extracted following the RNAeasy (Qiagen, Valencia, CA, USA) manufacture's protocol. Total RNA (100 ng) was used to synthesize cDNA using a qScript cDNA Supermix kit (Quanta Biosciences, Gaithersburg, MD, USA). Quantification of expression for TF and 18S were performed in a LightCycler 480 (Roche Applied Science, Indianapolis, IN, USA) using a PerfeCTa® SYBR® Green FastMix® (Quanta Biosciences) and specific primers for TF [67] or 18S housekeeping gene. Relative quantification analysis of TF versus 18S was performed using LightCycler 480 software [68].

### ELISA for IL-6, IL-8 and MCP-1

MVECs were incubated overnight with Tempol (0, 1, and 3 mM) followed by addition of LPS (200 ng/mL in EBM-2 Plus) for six hours as described above. The supernatant was collected and centrifuged, and the concentration of IL-6, IL-8, and MCP-1 was determined by ELISA using reference standard curves prepared with known amounts of recombinant cytokines, according to manufacturer's instructions (R&D Systems). Supernatant was diluted 1∶2 for IL-6, 1∶4 for MCP-1, and not diluted for IL-8 measurements.

### Cell Culture and Differentiation

HL-60 cells were maintained in an undifferentiated state in RPMI 1640 media containing 10% fetal bovine serum and 25 mM HEPES at 37°C in a humidified 5% CO_2_ atmosphere. HL-60 cells differentiated in culture medium containing 1.3% DMSO for five days before experiment. Cells were incubated for 3 hours at 37°C with Tempol.

### EZ-TAXIScan Chemotaxis Assay

The EZ-TAXIScan chamber (Effector Cell Institute, Tokyo, Japan) was assembled as described by the manufacturer [69]. Cell migration was recorded every 15 seconds for 30 minutes at 37°C in a humidified environmental chamber. Coverslips and chips used in the chamber were coated with 1% BSA at RT for one hour. All glass coverslips were ultrasonicated and washed before use. Cell migration analysis was conducted with DIAS software.

### ROS Detection

Differentiated HL-60 cells were collected and washed twice with Hank's balanced salt solution (HBSS; Invitrogen). Cells (2×10^6^/mL, 50 µL) were treated for 15 minutes at 37°C with or without 10 µM DPI chloride (Sigma-Aldrich). Cells were stimulated with 1 µM fMLP. Extracellular O_2_^−^ production was measured as SOD-inhibitable chemiluminescence detected using Diogenes reagent (National Diagnostics, Atlanta, GA, USA) using a 96-well plate Luminoskan luminometer (Thermo, Waltham, MA, USA). Extracellular H_2_O_2_ was measured by a luminol/HRP-based chemiluminescence assay. Briefly, cells (2×10^6^/mL, 50 µL) were collected as above and treated with or without 10 µM DPI (37°C for ten minutes). An equal volume of HBSS containing 1 mM luminol and 20 U/mL HRP was added following DPI treatment. Luminescence was estimated as above. Superoxide production and H_2_O_2_ production was presented as integrated luminescence value (relative light unit) [70].

### Platelet Preparation and Aggregation

Blood samples were obtained from paid healthy volunteers who gave written informed consent to participate in an IRB-approved study for the collection of blood samples for in vitro research use. The protocol is designed to protect subjects from research risks as defined in 45CFR46 and to abide by all NIH guidelines for human subjects research (protocol number 99-CC-0168). Human platelet-rich plasma was obtained by plateletpheresis at the NIH Department of Transfusion Medicine under the direction of Dr. Susan Leitman. Platelets were incubated with Tempol (0, 1 and 3 mM) for three hours at RT, then diluted to 2×10^5^ platelets/µL in Tyrode's buffer (137 mM NaCl, 2 mM KCl, 0.3 mM NaH_2_PO_4_, 12 mM NaHCO_3_, 5.5 mM glucose, 0.35% BSA, 1 mM MgCl_2_, and pH 7.4) [70]. Three hundred µL of platelets were added to the cuvettes, and 1 µg/mL Horm collagen was used as an agonist. To estimate granule secretion, Luciferin-luciferase reagent was added four minutes after addition of collagen. Platelet aggregation (detected by a change in transmission) and adenosine triphosphate (ATP) secretion (estimated by peak of luminescence) was monitored in a Chrono-Lumi platelet aggregometer (Chrono-Log) with continuous stirring (1200 rpm) at 37°C [70].

### Maturation and Function of DCs

Bone marrow-derived DCs from BALB/c mice were generated in the presence of recombinant murine GM-CSF (BioLegend, San Diego, CA, USA) as previously described [71], [72]. After six days of culture, cells were collected and washed, and a suspension containing 10^6^ cells/mL was prepared. For maturation assays, DCs were preincubated overnight with medium only or Tempol (0.3, 1, and 3 mM), followed by stimulation with ultrapure LPS (200 ng/mL). After 24 hours, maturation markers were evaluated in CD11c^+^ cells by flow cytometry as described [71]. Briefly, cells were incubated with fluorochrome-labeled monoclonal antibodies to CD11c, CD40, CD80, CD86, and MHC class II (I-A/I-E), and a total of 100,000 events were acquired using a FACSCanto II (BD Biosciences). Analysis was performed using FlowJo software, version 7.5.5 (Tree Star, Ashland, OR, USA). For evaluation of DC function, cells were preincubated overnight with medium or Tempol (3 mM) and pulsed with OVA (100 µg/mL) plus LPS (200 ng/mL) for four hours. After three washings to remove all stimuli, DCs (25,000) were co-cultured for 72 hours in 96-well plates in the presence of CD4^+^ T cells (100,000) purified from spleens of congenic DO11.10 mice using microbeads and MACS columns (Miltenyi Biotec Inc., Auburn, CA, USA). DO11.10 mice express a transgenic TCR specific for the peptide 323–339 generated from OVA processing by DCs. Resazurin sodium 0.01% (in culture medium) was added at 10% of incubation volume in the last 24 hours of culture. Cell proliferation was evaluated by reading the absorbance of each well at 570 and 600 nm, as previously described [71]. At the end of the culture, plates were centrifuged and cell-free supernatants collected for IL-2 and IFN-γ determination according to manufacturer's instructions (BD Biosciences).

### Culture of *P. falciparum* Parasites

Mycoplasma-free parasites (3D7) were thawed and initially grown in a 5% suspension of purified human O^+^ RBCs in RPMI 1640 medium supplemented with 10% human serum, 2 g/L sodium bicarbonate, 0.1 mM hypoxanthine, 25 mM Hepes (pH 7.4), and 10 mg/L gentamicin at 37°C, 5% O_2_, 5% CO_2_, 90% N_2_. Cells were synchronized biweekly by sorbitol treatment. For each experiment, late-stage (mature) parasites identified by microscopy as late trophozoites or schizonts were isolated on a 70/40% Percoll-sorbitol gradient. This resulted in parasitemia of 97% to 99%. Parasites were counted and diluted with uninfected RBCs in culture medium to <1.5% late-stage parasitemia at 2% hematocrit unless specified otherwise. For membrane feeding assays, parasites were grown in a 5% suspension of human O+ RBCs in RPMI 1640 medium supplemented with 10% human serum, 2 g/L sodium bicarbonate, 0.1 mM hypoxanthine, 25 mM Hepes (pH 7.4), at 37°C, 5% O_2_, 5% CO_2_, 90% N_2_.

### Growth Inhibition Assays of 3D7 Parasites

Synchronous *P. falciparum* parasite cultures were grown in RPMI 1640 medium (Invitrogen) supplemented with 10% human serum, 2 mg/mL sodium bicarbonate (Gibco, Invitrogen), 0.10 mM hypoxanthine (Sigma-Aldrich), 25 mM HEPES pH 7.4 (Calbiochem, EMD Chemicals, Inc.), 10 mg/L Gentamicin (Gibco, Invitrogen) and incubated at 37°C in a 5% O_2_, 5% CO_2_, 90% N_2_ atmosphere. Antioxidants were added at different concentrations directly to *P. falciparum* cultures containing 0.03% trophozoite-stage parasites. After 48 hours of culture, the respective numbers of trophozoites were determined by spectrophotometric detection of parasite LDH (pLDH assay) as described [73], [74]. One hundred percent inhibition was set as unparasitized RBCs and 0% inhibition as parasitized RBCs. The stocks for each antioxidant were: Tempol (10 mM in RPMI), PBN (100 mM in DMSO), MnTE-2-PyP (10 mM in RPMI), MnTBAP (10 mM in RPMI), Mitotempo (95 mM in DMSO), MitoQ (15 mM in ethanol), M30 (25 mM in DMSO), epigallocatechin (10.9 mM in RPMI), and DPI (31.6 mM in DMSO). Controls were run in parallel, and no inhibition was observed with 0.4% DMSO (maximum final concentrations in the assay).

### Animals, Malaria Infections, and Treatment with Tempol, other Antioxidants, DETC, and DPI

Female 11-week-old C57BL/6 mice (20 grams) were obtained from Charles River Laboratories International Inc. (Frederick, MD, USA) and allowed to acclimate in the animal facility until the experiment was performed. Parasites were obtained from donor mice infected with thawed parasite stocks. Mice were intraperitoneally (i.p.) infected with 10^6^ *Plasmodium berghei* Anka strain pRBCs. Parasitemias in infected mice were quantified by examining Giemsa-stained blood smears daily. Hemoglobin levels were measured every other day using an Hp201+ (HemoCue, Ängelholm, Sweden) using blood from the tail tip. All mice were evaluated daily for the presence of clinical signs of severe malaria using simple scoring adapted from the SNAP scoring system [26], [75], [76]. Animals were scored by evaluating five categories: interactions, cage grasp, visual placing, gait/posture/appearance, and capacity to hold their body weight on a baton [75]. Each category was divided into three levels, varying from 0 to 2, where 0 represented normal individuals and 2 the worst case for the particular parameter as described in detail [26]. For treatments, mice were injected i.p. once daily with 100 µL Tempol (stock solution 4 mg/mL in saline, 20 mg/kg; 10 animals/group x 4; total 40 mice) [57], [77], 100 µL MnTE-2 PyP (stock solution 1 mg/mL in saline, 5 mg/kg; 10 animals/group) [78], 100 µL PBN (stock solution 10 mg/mL in 1.6% DMSO, 50 mg/kg; 10 animals/group) [79], 100 µL Epigallocatechin gallate (EGCG, stock solution 5 mg/mL in saline, 25 mg/kg; 10 animals/group) [80], 100 µL of M30 (stock solution 1.5 mg/mL in 1.6% DMSO, 7.5 mg/kg; 10 animals/group) [81], 100 µL Mitotempo (stock solution 50 mg/mL in DMSO, then diluted to 0.6 mg/mL in saline, 3 mg/Kg; 10 animals/group) [82], MitoQ (stock solution 60 mg/ml in ethanol then diluted to a 2 mg/ml solution containing 5%PEG400, 5% Tween 80 and 4% ethanol, 10 mg/Kg, 10 animals/group)[83], [84], and 100 µL MnTBAP (stock solution 2 mg/mL in saline, 10 mg/Kg; 10 animals/group) [85]. For some experiments, 100 µl of DETC (stock solution 10 mg/mL in saline, 50 mg/kg; 10 animals/group), a known SOD1 inhibitor [86] or 100 µL DPI (stock solution 5 mg/mL in DMSO and warmed at 45°C, then diluted in saline to 0.4 mg/mL saline, 2 mg/Kg; 10 animals/group) [87]–[90] were injected once daily (i.p.) starting at day 1 post infection (p.i.).

As controls, infected mice were injected with 100 µL of saline or 100 µL of the corresponding vehicle. Treatment started day 1 or day 4 p.i. and continued thereafter until animals became moribund, when the protocol was interrupted. Otherwise, animals were followed for 15 days and euthanized. For experiments where blood samples and brain tissue were collected, the exact same protocol and number of mice (10/group) described above was performed; however, on day 6 p.i. (a time point when all animals were alive), blood was drawn from the mandibular plexus. To estimate complete blood count, blood (≈30 µL) was collected in EDTA KE/1.3 tubes (Sarstedt, Newton, NC, USA) and evaluated with a Hemavet 950FS (Drew Scientific Inc, Oxford, CT, USA). For quantification of serum cytokines, blood (≈200 µL) was collected in serum gel Z/1.1 tubes (Sarstedt). After 20 minutes at RT, the Eppendorfs were centrifuged (five minutes at 5,000×*g* at RT) and serum collected, transferred to another Eppendorf, and frozen. Quantification of MCP-1, IFN-γ, TNF-α, IL-2, IL-6, and IL-10 were carried out at Quansys Biosciences (Logan, UT, USA). To obtain tissue for pathology studies, the animals were euthanized in a CO_2_ chamber and their brains immediately removed from the skulls and placed in formalin 10% for fixation. All in vivo experiments were approved by the NIAID Internal Review Board and followed the rules for animal experimentation; this protocol was annotated as LIG-1E, under the directions of Dr. Ted Torrey (NIAID/NIH).

### Brain Pathology

Using MultiBrain® technology (NeuroScience Associates, Knoxville, TN, USA), 20 mouse brains were embedded together per block (5 brains for each of four groups: uninfected, infected treated with saline, infected treated with Tempol day 1, or infected treated with Tempol day 4) for a total of 40 brains. The blocks were freeze-sectioned at 30 C in the sagittal plane through the entire mouse brain (≈9 mm in length) at NeuroScience Associates. Blood brain barrier (BBB) compromise stain detects IgG in the brain parenchyma and reveals increased permeability of the BBB. Each brain was sectioned at 720-µm intervals, yielding 13 slides per block, or 520 stained total for all 40 brains. The presence of brain parenchyma IgG indicates extravasation of the blood, which is a marker of increased permeability of the BBB.

### Experiments with Knockout (KO) Mice

Mice deficient in phagocyte NOX2 (NADPH oxidase, gp91^phox–/–^; strain B6.129S-Cybbtm1Din/J; #002365) and age- and gender-matched control C57BL/6J (# 000664), or deficient in mitochondria superoxide dismutase 1 (SOD1) (strain B6;129S-Sod1tm1Leb/J, #002972) and age- and gender-matched B6129SF2/J control (#101045) were purchased from Jackson Laboratory (Bar Harbor, ME, USA). KO or control mice (n = 8/group) were infected by 10^6^ *P. berghei* and evaluated for clinical scores and survival as described above. On day 6 p.i., an aliquot of blood was obtained to estimate parasitemia as above.

### Statistical Analysis

Results are expressed as means ± SEM. Significance was set at P≤0.05. Statistical differences among the groups were analyzed by analysis of variance using Tukey or Bonferroni as a multiple comparison post-test. Kaplan-Meyer plots were used for survival curves and statistical analysis performed with the log-rank test.

## Results

### Tempol Inhibits Expression of TF and Cytokine Generation in LPS-Stimulated ECs

**Figure 1:**
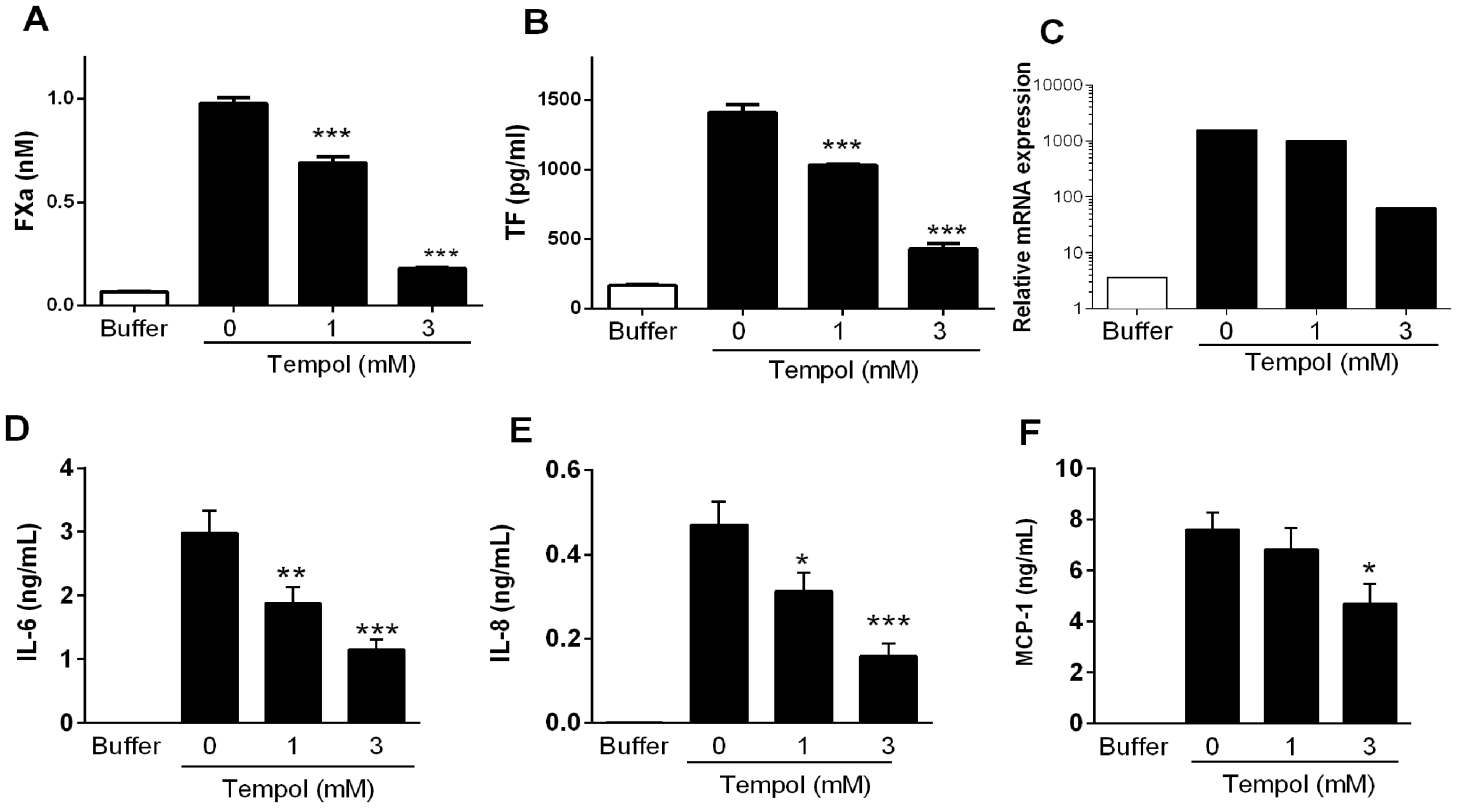
Tempol inhibits transcription and functional expression of tissue factor in endothelial cells, and cytokine production. A, Inhibition of tissue factor (TF) activity. MVEC were incubated overnight with Tempol (0, 1, and 3 mM) followed by washing the wells and addition of lipopolysaccharide (LPS) (200 ng/mL). A mixture containing FX and FVIIa was then added to the cells, and FXa generation was estimated in the supernatant using chromogenic substrate (S2222), as described in Materials and Methods. B, Inhibition of TF generation. Cells were incubated overnight with Tempol (0, 1, and 3 mM) followed by washing of the wells and addition of LPS (200 ng/mL) for six hours. Wells were washed and cells were lysed with 0.1% Triton X-100. The supernatant was used to detect TF antigen by ELISA. C, Inhibition of TF transcription. Cells were incubated overnight with Tempol (0, 1, and 3 mM) followed by addition of LPS (200 ng/mL) for 2 h. Cells were washed and trypsinized for extraction of mRNA. Real-time PCR of the samples was evaluated as described in Materials and Methods. The figure shows a representative result from two independent experiments. D–F, Inhibition of cytokine generation. MVEC were incubated overnight with Tempol (0, 1, and 3 mM) followed by washing of the wells and addition of LPS (200 ng/mL). After six hours, the supernatant of the cells was collected and used for detection of D, IL-6, E, IL-8, and F, MCP-1 by ELISA (n = 8). *, P≤0.05 (analysis of variance, Bonferroni post-test).

Although antioxidants have been tested as inhibitors of TF expression [91], the potential effects of Tempol as a modulator of coagulation have remained elusive thus far. To determine whether Tempol affects expression of TF in ECs, a monolayer of cells in culture was incubated with the drug overnight, followed by washing and addition of LPS (200 ng/mL) for six hours. LPS was used as a ligand to activate TLR. A mixture of FVIIa/FX was then added, and FXa generation was used as a “read-out” for TF expression. Experiments performed side by side show that Tempol (0–3 mM) dose-dependently inhibits functional expression of TF induced by LPS with IC_50_ between 1 and 3 mM (Figure 1A). Tempol also inhibits ≈35% TF expression induced by TNF-α (not shown), a cytokine that is increased in *P. falciparum* infection [1]. Next, experiments were performed to determine whether Tempol prevents production of TF induced by LPS. Figure 1B demonstrates that Tempol inhibits TF generation (IC_50_ around 2 mM), determined by ELISA for TF. Tempol also partially (30%) prevents TNF-α-induced generation of TF (not shown). Regulation of TF gene is under control of several transcriptional factors including AP-1, Egr-1, and NF-κB [92]. Experiments performed with antioxidant succinobucol (AGI-1067) determined that ROS play a role in LPS induction of nuclear AP-1 and Egr-1 [91]. To determine whether Tempol affects transcription of TF, cells previously treated with Tempol were stimulated with LPS for 90 minutes and the mRNA extracted for real-time PCR assays. Figure 1C shows that LPS induces strong mRNA expression for TF in ECs by a mechanism that was concentration-dependently inhibited by Tempol. To ascertain whether Tempol inhibits generation of pro-inflammatory cytokines, ECs were treated with Tempol as above and—after six hours of stimulation with LPS—the supernatant was collected and tested for cytokine generation by ELISA. Figures 1D, E, and F demonstrate that Tempol reduced the production of IL-6, IL-8, and MCP-1, respectively. These results provide direct evidence that Tempol interferes with an early event associated with TF expression and production of inflammatory cytokine.

### Tempol Inhibits ROS Generation by HL-60 Cells Stimulated by *f*MLP and Platelet Aggregation Induced by Collagen

**Figure 2:**
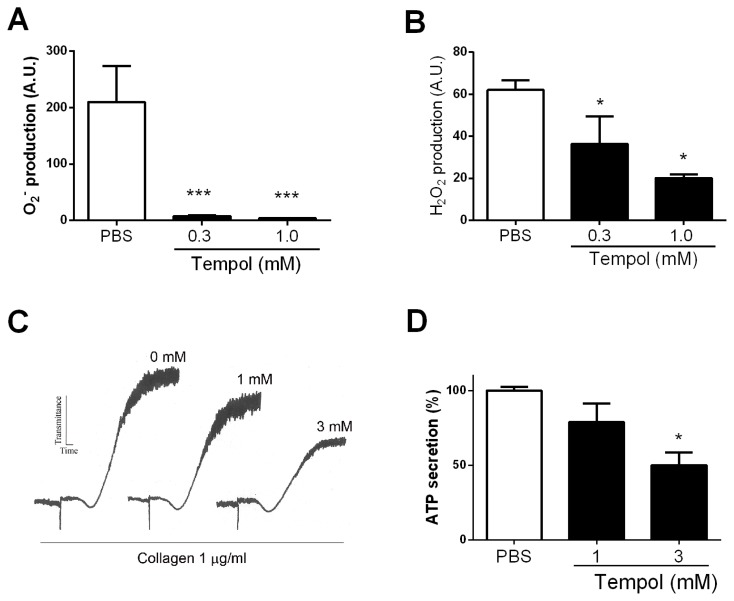
Tempol inhibits neutrophil and platelet pro-hemostatic functions. Inhibition of superoxide production. HL-60 cells were incubated with Tempol (0, 0.3, and 1 mM) followed by addition of *f*MLP (1 µM). Superoxide production was detected with Diogenes reagents. **B,** Inhibition of H_2_O_2_ production. HL-60 cells were incubated with Tempol (0, 0.3, and 1 mM) followed by addition of *f*MLP (1 µM). H_2_O_2_ production was detected with luminol/HRP-based chemiluminescence assay. **C,** Effects on platelet aggregation. Platelet-rich plasma was incubated with Tempol (0, 1, and 3 mM) for three hours and activated with collagen (1 µg/mL). A representative tracing is shown (similar results obtained with two other donors). **D,** Effects on platelet secretion. Platelets were activated as above, and ATP was determined by luminescence using Luciferin-luciferase reagent. Experiments were performed in triplicate or quadruplicate. *, P≤0.05 (analysis of variance, Bonferroni post-test).

Neutrophils play a major role in vascular inflammation through a number of mechanisms including ROS generation. In malaria, heme release potentially activates neutrophils that, in turn, release elastases, which exhibit pro-inflammatory and pro-coagulant properties [93], [94]. To verify the effects of Tempol in ROS production, HL-60 cells—which are used as a surrogate to study neutrophil function—were incubated with Tempol and activated with *f*MLP to stimulate oxidative burst. Figure 2A demonstrates that Tempol at a concentration of <0.3 mM completely abolishes O_2_^−^ detection; at 0.3 mM and higher; it also attenuates H_2_O_2_ levels triggered by *f*MLP (Figure 2B). These results validate the view that the drug is a potent and effective antioxidant. In contrast, Tempol did not affect *f*MLP-mediated chemotaxis of HL-60 cells (i.e. total path length, net path length, directionality, direction change, and speed) as determined with a TAXIscan assay (data not shown).

Platelets play a major role in malarial pathogenesis by promoting pRBC interaction with the endothelium, resulting in production of inflammatory cytokines or amplification of the coagulation cascade [4], [7]. Platelets are an important source for ROS [95], [96], and O_2_^−^ participates in the activation of platelets by collagen. Figure 2C demonstrates that Tempol, at 1 and 3 mM, partially but consistently inhibits platelet aggregation by collagen. Inhibition was characterized by a decrease in maximum amplitude response without effects in shape change. Tempol at similar concentrations attenuated granule secretion estimated by ATP release detected with Luciferin-luciferase reagent (Figure 2D).

### Tempol Inhibits DC Function

**Figure 3:**
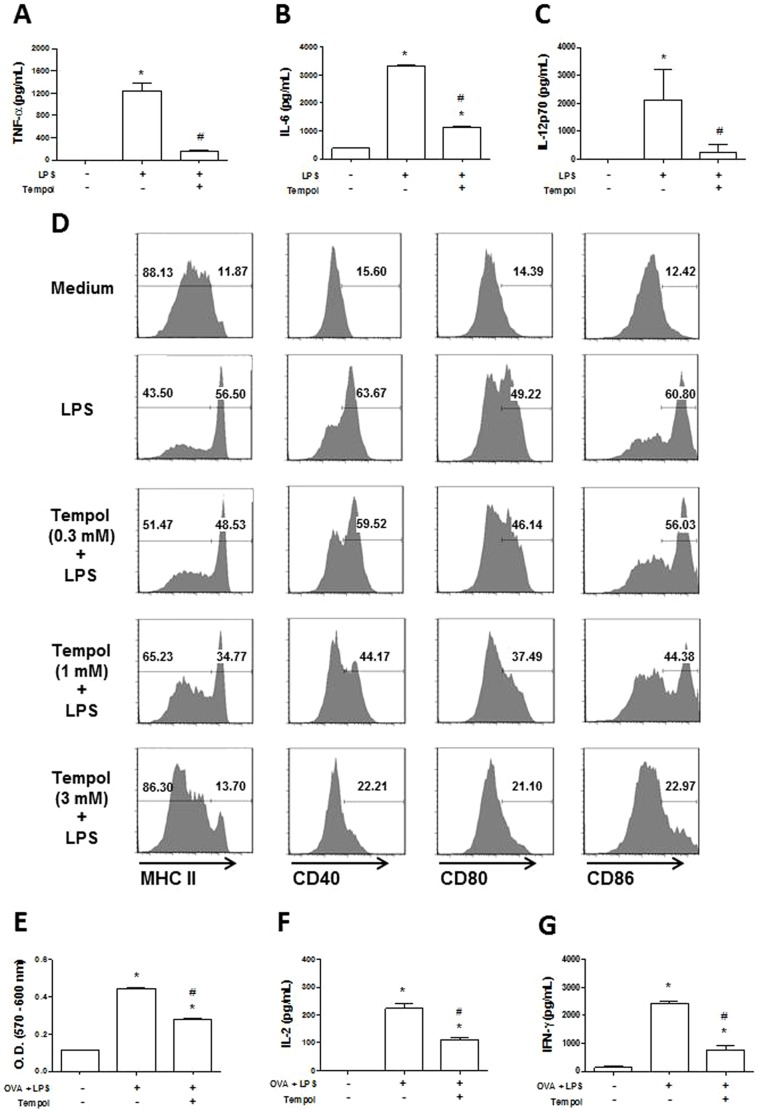
Tempol inhibits dendritic cell (DC) functions. Bone marrow-derived DCs (10^6^ cells/mL) were preincubated overnight with medium or Tempol at indicated concentrations and stimulated with lipopolysaccharide (LPS) (200 ng/mL) for 24 hours. Detection of **A,** TNF-α was evaluated in cell-free culture supernatants after six hours, and the levels of **B,** IL-6 and **C,** IL-12p70 were evaluated after 24 hours. **D,** Cells were stained with fluorochrome-labeled monoclonal antibodies to CD11c, CD40, CD80, CD86, and MHC class II and analyzed by flow cytometry. **E,** DCs incubated with medium or Tempol were pulsed with OVA (100 µg/mL) plus LPS (200 ng/mL) for four hours. After repeated washings, DCs were co-incubated with purified CD4^+^ cells from DO11.10 mice (DC:CD4^+^ ratio  = 1:4) for 72 hours, and proliferation was measured as described in Materials and Methods. **F,** IL-2 and **G**, IFN-γ were evaluated in the culture supernatants from the proliferation assay. *, P≤0.05 versus control group (^–/–^); ^#^, P≤0.05 versus LPS group (^+/−^) (analysis of variance).

DCs are important mediators of inflammation through production of inflammatory cytokines TNF-α and IL-12, which are critical for their maturation and function. DCs also participate in sustaining the coagulation response [38]. In malaria, *P. falciparum* releases *Pf*-GPI during schizogony, a lipid that activates TLR2/4 and contributes to inflammation and cytokinemia in malaria [26]. DC function is also modulated by ROS [97], and it was of interest to determine whether Tempol's anti-inflammatory mechanism of action could be explained— at least in part—by downmodulating DC biology. To that aim, DCs were incubated overnight with Tempol followed by addition of LPS as maturation stimulus. ELISA was used to determine production of cytokines by DCs, and flow cytometric analysis was employed to estimate the expression of co-stimulatory molecules as markers of DC maturation. Figures 3A, B, and C demonstrate that Tempol (3 mM) inhibits LPS-induced production of TNF-α, IL-6, and IL-12p70, respectively. Figure 3D shows the expression pattern of MHC II, CD40, CD80, and CD86 under basal conditions corresponding to 11.87%, 15.60%, 14.39%, and 12.42%. When LPS is added, the proportion of positive cells increases to 56.5% (MHC II), 63.67% (CD40), 49.22% (CD80), and 60.8% (CD86) and thus confirms successful maturation. Notably, the presence of Tempol in the cultures dose-dependently prevented expression of all co-stimulatory molecules with an IC_50_ ≈1 mM. These results suggest that ROS formation plays an active role in DC maturation by a mechanism that is inhibited by Tempol.

To verify whether this response was associated with interference in DC function, an assay was carried out to observe the intracellular processing of OVA as a model antigen and its presentation to CD4^+^-specific T cells. In this assay, T cells were genetically engineered to recognize OVA peptides in DC MHC molecules through their receptors; the “read-out” of the assay is T cell proliferation and cytokine production. Figure 3E demonstrates that Tempol (3 mM) significantly inhibits OVA-induced proliferation of T cells in the presence of DCs. This response was accompanied by inhibition of IL-2 (Figure 3F) and IFN-γ (Figure 3G) production. These results corroborate the view that downmodulation of O_2_^−^ production by Tempol is associated with diminished DC function.

### Tempol Inhibits Growth of *P. falciparum*

**Figure 4:**
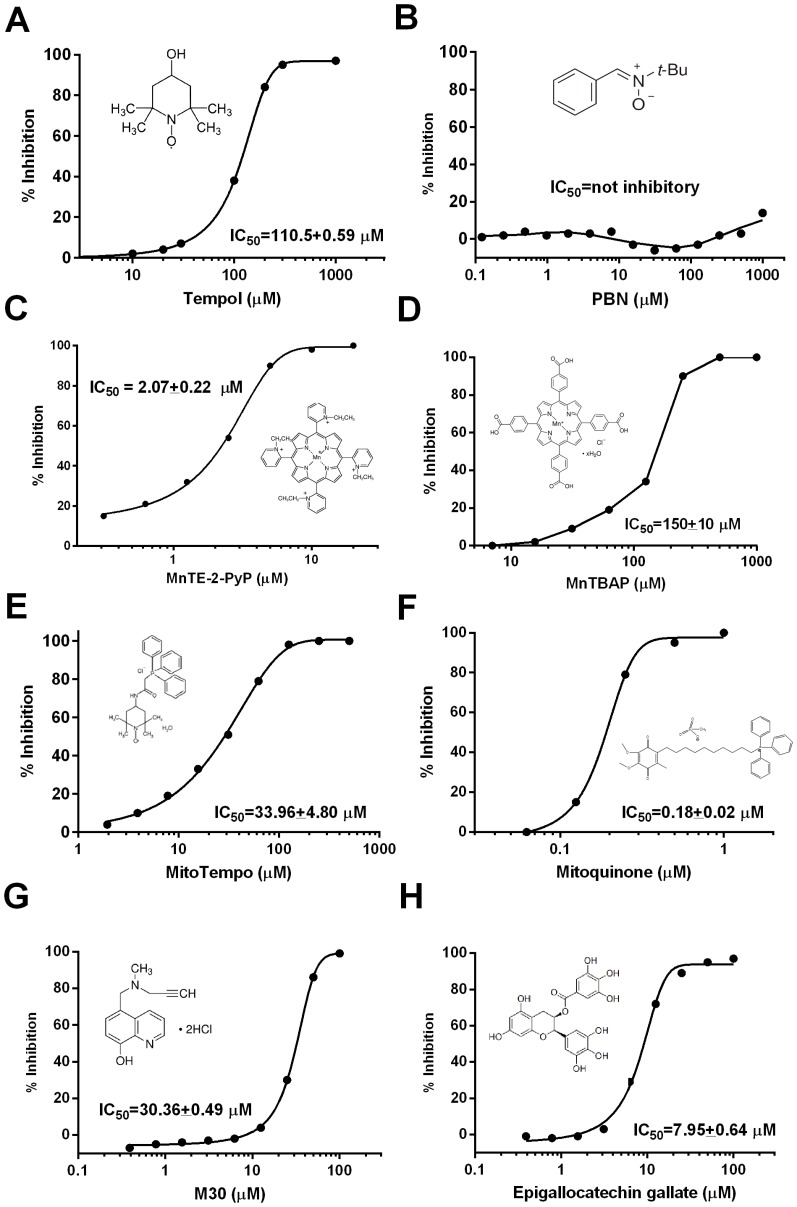
Tempol blocks parasite growth in vitro. Comparison with other antioxidants. **A,** Tempol; **B,** PBN; **C,** MnTE-2-PyP; **D,** MnTBAP; **E**, Mitotempo; **F**, Mitoquinone; **G**, M30; and **H,** Epigallocatechin gallate were added at the indicated concentrations directly to *P. falciparum* cultures containing trophozoite-stage parasites. After 48 hours of culture, the respective number of trophozoites present in the cultures was determined by pLDH (growth inhibition assay). Stock solution for all reagents was diluted in the culture assay media, or as specified in Materials and Methods. The chemical structure of each compound, and the corresponding IC_50s_, are shown as an inset.

Tempol was tested in the life cycle of *P. falciparum*. Tempol added to a parasite culture enriched with late trophozoites and schizonts prevents parasite growth in vitro with an IC_50_ ≈110 µg/mL (Figure 4A). By contrast, no effect was observed when PBN—another spin trap and antioxidant—was added to culture (Figure 4B). Notably, MnTE-2-PyP, a porphyrin-based antioxidant, exhibited a remarkable effect in the growth inhibition assay, with an IC_50_ ≈2 µM (Figure 4C). In contrast, MnTBAP— another well studied porphyrin-based antioxidant—attenuated parasite growth with IC_50_ ≈150 µM (Figure 4D). The mitochondria-directed antioxidants Mitotempo (Figure 4E) and MitoQ (Figure 4F) inhibited parasite growth at IC_50_ ≈35 µM and ≈0.2 µM, respectively. Our results also show that M30 (an iron chelator) (Figure 4G) and EGCG (the green tea antioxidant) (Figure 4H) affected parasite growth with IC_50_ ≈30 µM and ≈8 µM, respectively. NADPH oxidase inhibitor DPI also inhibited growth, with low IC_50_ 0.29±0.021 µM (curve not shown; triplicates).

### Tempol Increases Survival of Mice Infected with *P. berghei* Anka strain

**Figure 5:**
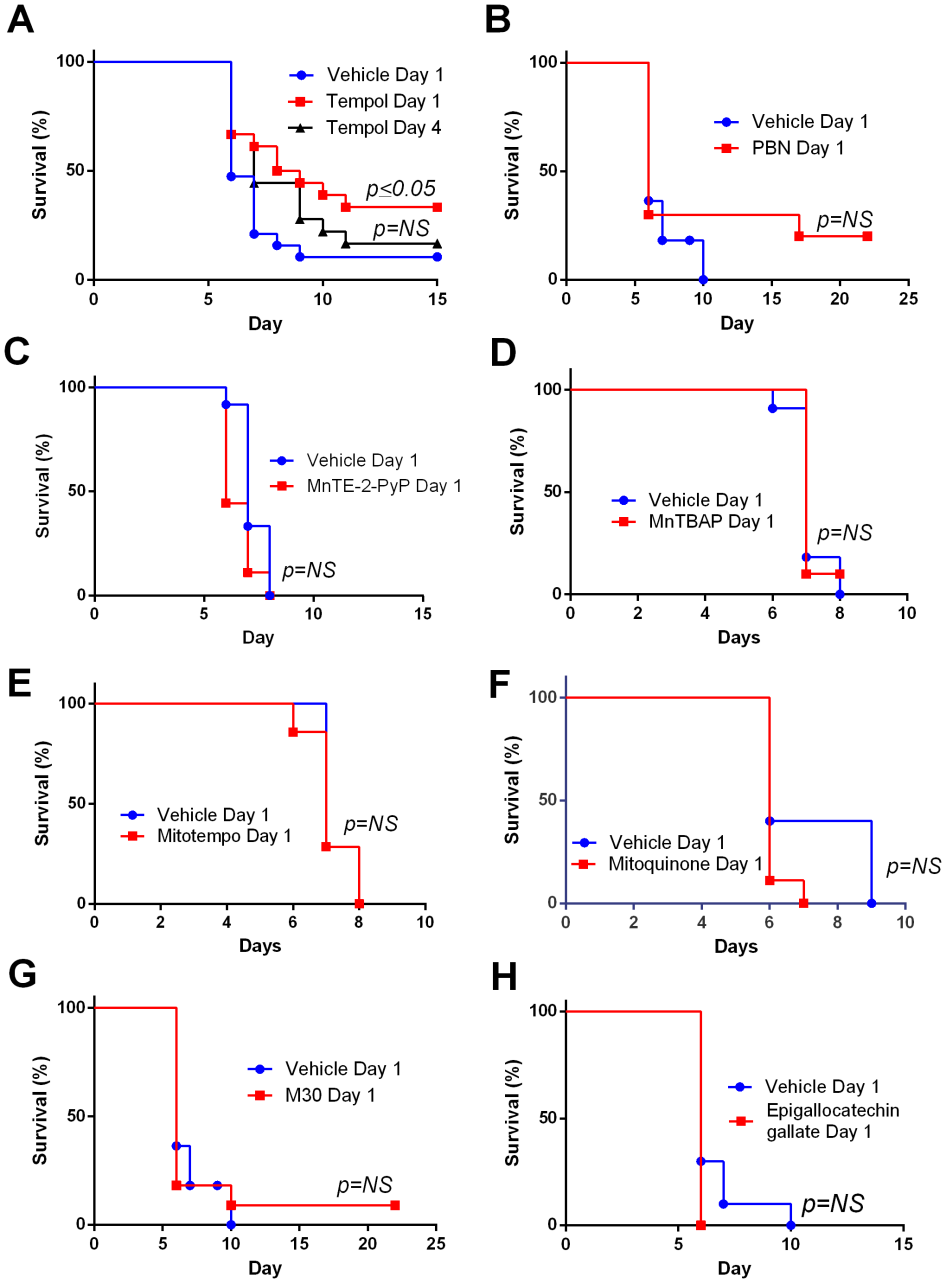
Tempol increases survival of mice in a cerebral malaria model. Comparison with other antioxidants. *Plasmodium berghei* Anka strain parasitized red blood cells (10^6^) were injected in mice intraperitoneally (i.p.). Then, **A,** Tempol (20 mg/kg, n = 40), **B,** PBN (50 mg/kg, n = 10), **C,** MnTE-2-PyP (5 mg/kg, n = 10), **D**, MnTBAP (10 mg/Kg, n = 10), **E**, Mitotempo (3 mg/Kg, n = 10), **F**, Mitoquinone (10 mg/Kg, n = 10), **G,** M30 (7.5 mg/kg, n = 10), and **H,** Epigallocatechin gallate (25 mg/kg, n = 10), were injected i.p. starting from day 1 (all reagents) or day 4 p.i. (for Tempol only) as indicated. One hundred µL of each antioxidant was injected i.p. daily/mice. The percent of non-treated and treated mice that survived over time is given in Kaplan–Meier curves. *, P≤0.05 (Log-rank test). NS, non-significant.

Effects of Tempol were evaluated in a murine model for CM. Groups of 10 mice were inoculated i.p. with 10^6^ *P. berghei* Anka-infected RBCs (pRBC) and followed for 15 days. Tempol was injected daily i.p. with 100 µL of a 4-mg/mL solution (20 mg/kg, n = 40) starting on day 1 or day 4 p.i. and continued thereafter. As a control, saline was injected in another group of infected animals. Most animals became sick with symptoms compatible with CM (e.g. lethargy, ataxia, lack of response to touch) at day 6, resulting in death on day 6 or 7. When Tempol administration started on day 1, some animals died at the same days as saline-treated animals; however, other animals presented delay in onset of neurologic symptoms as well as a reduction in the intensity of clinical signs (Figure 5A). Nonetheless, these animals were not as healthy as controls, and gradually became sick and eventually died, but days after most infected untreated (controls) animals have died. While this effect was consistent in all different experiments (10 animals/group), a statistically significant increase in survival (P≤0.05) was only observed when the data from four independent experiments were pooled. When Tempol was initiated on day 4 p.i., a small percentage of animals also survived longer than saline-treated groups, resulting in a trend for increased survival that did not reach the conventional threshold of significance (P≤0.05)(Figure 5A). Treatment with Tempol (30 mg/kg or 60 mg/kg) did not result in improvement in survival (data not shown; n = 10/group).

Next, other antioxidants that exhibit distinct mechanisms of action were tested for their effect on survival. Treatment with all antioxidants was started at day 1 p.i.. Figure 5B shows that PBN (10 mg/kg)—another spin trap and antioxidant [79]—was found to promote a trend for increase in survival. MnTE-2-PyP, a potent and well-studied SOD-mimetic [58], was tested at 5 mg/kg. Surprisingly, MnTE-2-PyP did not show any protective effect (Figure 5C). To the contrary, MnTE-2-PyP produced toxic effects five minutes after each i.p. injection after day 4 of therapy. Toxicity was characterized by a transient tremor and loss of balance lasting about 20 minutes (without temperature changes). The loss of balance was observed when animals were suspended by the tail; they spun frenetically. These effects were transient, lasting less than 30 minutes, and were observed in infected mice only, suggesting that CM lowers the toxicity threshold of MnTE-2-PyP. Likewise, MnTBAP (10 mg/Kg)—another porphyrin-based antioxidant [85]—was ineffective but did not cause neurologic manifestations upon injection (Figure 5D). Antioxidants that accumulated in the mitochondria, Mitotempo (3 mg/Kg) [82] and MitoQ (10 mg/Kg) [84], did not improve survival (Figures 5E and 5F, respectively). Also, M30 (an iron-chelator) [81], was given to mice at 7.5 mg/kg and was devoid of protective properties (Figure 5G). Finally, EGCG (25 mg/Kg), the major antioxidant from green tea [80], was ineffective in the CM model (Figure 5H). With the exception of MnTE-2-PyP, none of the antioxidants tested affected parasitemia in vivo (data not shown).

### Effects of Tempol in Clinical Score and Plasma Cytokine Levels

**Figure 6:**
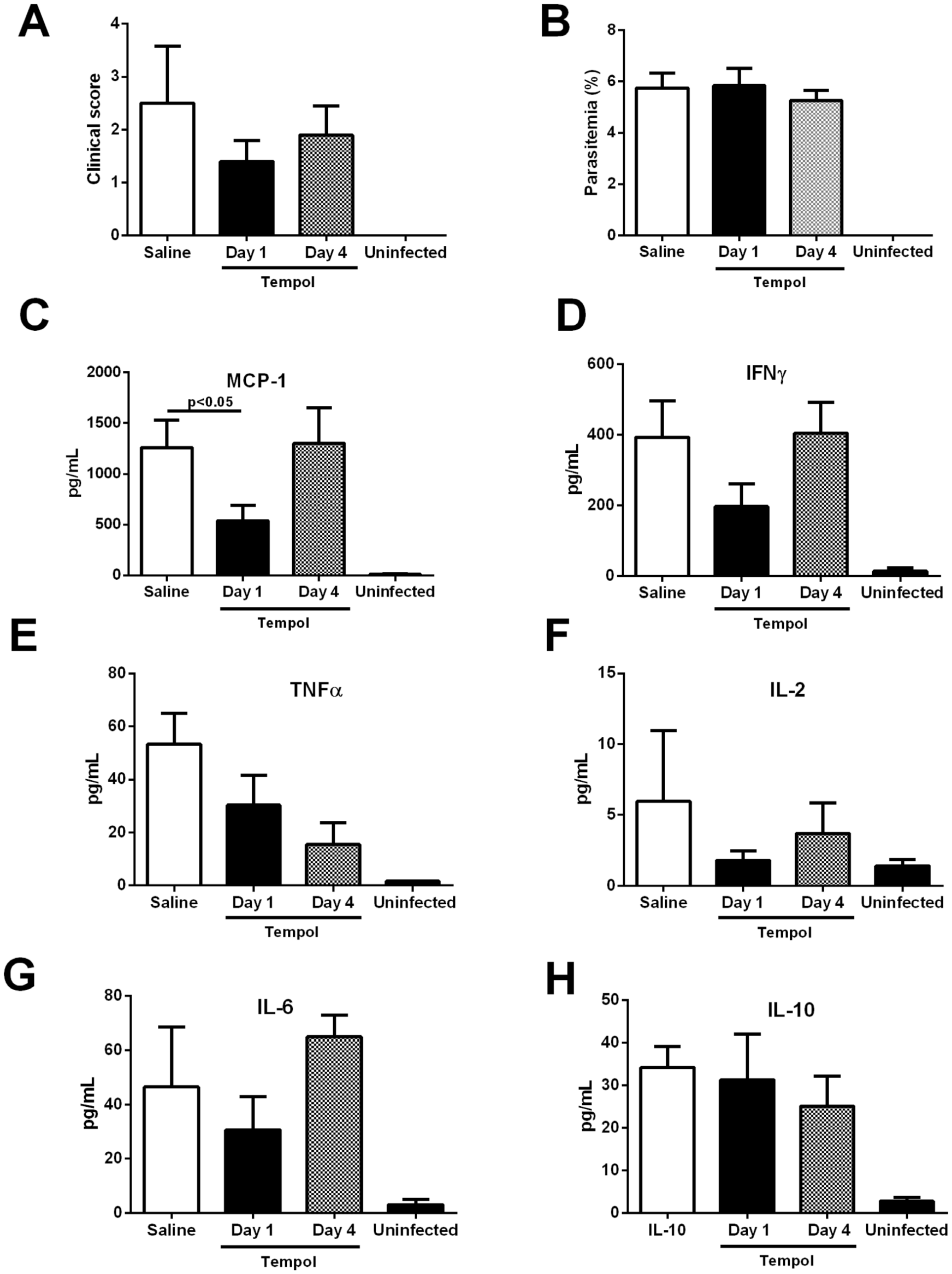
Tempol inhibits MCP-1 plasma levels of mice infected with *Plasmodium berghei* Anka. *P. berghei* Anka parasitized red blood cells (10^6^) (*n* = 10) were injected intraperitoneally (i.p.). **A,** Tempol (20 mg/kg), was started at day 1 or day 4 post infection (p.i.). At day 6 p.i., several parameters were determined including **A,** clinical score and **B,** parasitemia (determined by Giemsa-stained smears of tail blood). Serum was collected for determination of **C,** MCP-1, **D,** IFN-γ, **E,** TNF-α, **F,** IL-2, **G,** IL-6, and **H,** IL-10 by enzyme-linked immunosorbent assay as described in Materials and Methods. *, P≤0.05 (analysis of variance, Tukey post test). NS, non-significant. Ten mice were analyzed per group.

We examined whether Tempol administration could affect the level of inflammatory cytokines when therapy started at day 1 or day 4. Groups of 10 mice were infected, and blood samples for all animals were collected at day 6, when most animals were still alive. Figure 6A shows that infection with *P. berghei* Anka is accompanied by a trend for amelioration of the clinical score. All animals exhibited the same parasitemia (Figure 6B), indicating that Tempol was devoid of in vivo antimalarial effects. Figures 6C–H show that infection increases serum levels of several cytokines including MCP-1, IFN-γ, TNF-α, IL-2, IL-6, and IL-10. Tempol produced a significant decrease in the levels of MCP-1 (P≤0.05), with a trend for lower levels for IFN-γ (Figure 6D), IL-2 (Figure 6F), and IL-6 (Figure 6G). No changes were observed for TNF-α (Figure 6E) or IL-10 (Figure 6H).

### Effects of Tempol in Hematologic Parameters

Mice treated with Tempol starting at day 1 p.i. also did not display changes in RBC numbers, hemoglobin, hematocrit levels, platelets, white blood cells, and lymphocyte numbers (data not shown).

### Tempol Interferes with BBB Permeability

**Figure 7:**
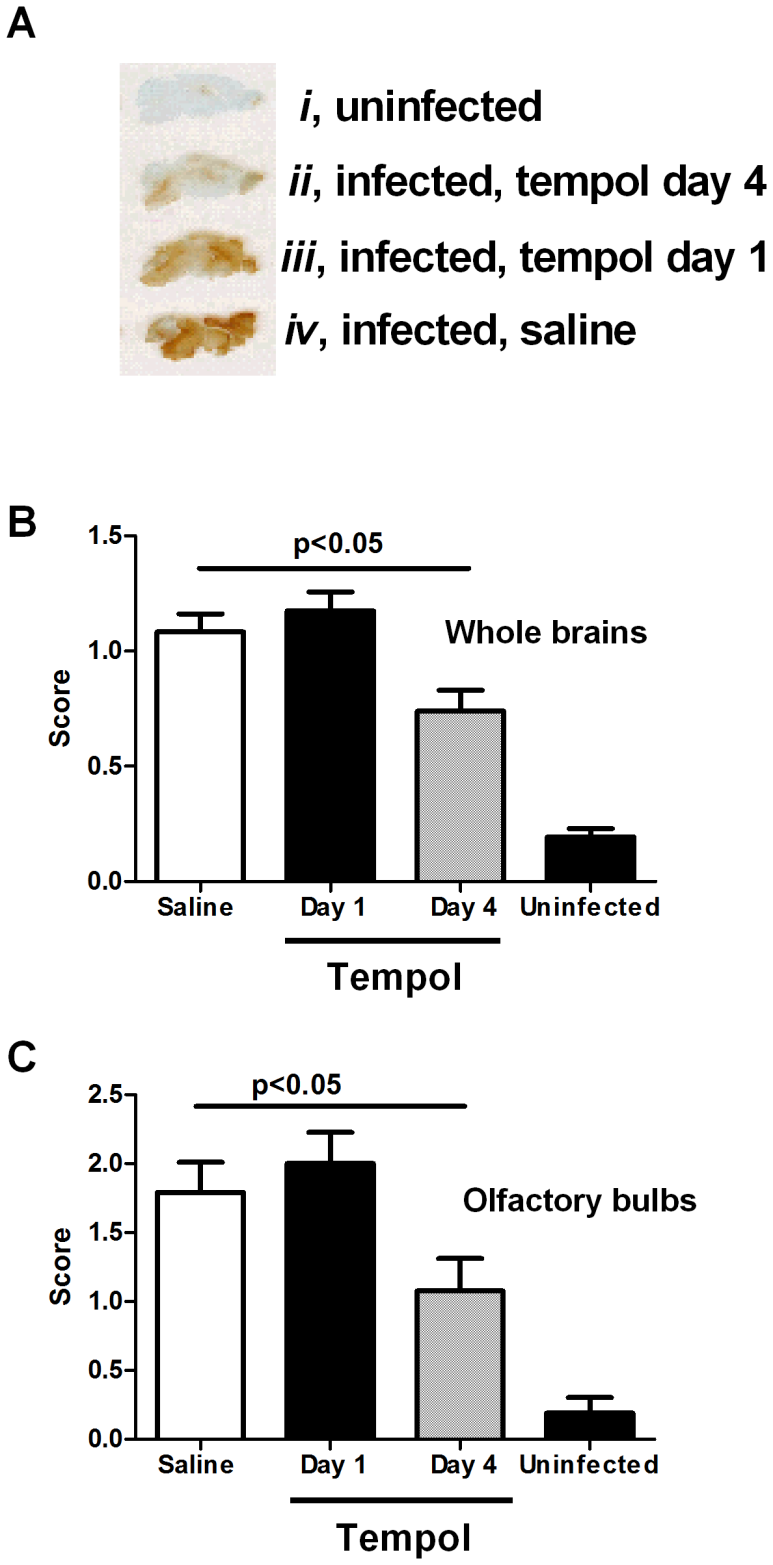
Tempol inhibits increase of permeability of the blood brain barrier (BBB). *Plasmodium berghei* Anka parasitized red blood cells (10^6^) were injected into mice intraperitoneally (i.p.) Tempol (100 µL; 20 mg/kg), was started at day 1 or day 4 post infection (p.i.). At day 6 p.i., all animals were euthanized and the brain immediately dissected and fixed. Permeability of the BBB was detected as extravasation of IgG as described in Materials and Methods. **A,** A typical experiment is shown: ***i,*** uninfected mice; ***ii,*** infected and treated with Tempol (day 4); ***iii,*** infected and treated with Tempol (day 1); and ***iv,*** infected, non-treated mice. **B,** Quantification of inflammation of the whole brain. **C,** Quantification of inflammation of the olfactory bulbs. Quantification was performed as described in Materials and Methods. Ten mice were analyzed per group. *, P≤0.05 (ANOVA, Tukey post-test).

In an attempt to determine whether therapeutic targeting of O_2_^−^ in vivo with Tempol was associated with protection against CM-associated pathologic changes in the brain, brains were obtained on day 6 p.i. and were tested for an increase in BBB permeability by staining with anti-mouse IgG and scoring according to the scale shown on Figure 7A. On average, the BBB in uninfected mice exhibits no IgG extravasation, indicating the absence of changes in permeability. Non-treated infected animals presented strong staining, representing extravasation of IgG in the parenchyma. Mice receiving Tempol treatment starting on day 4 p.i. exhibited dramatically reduced BBB permeability. In contrast, when Tempol was started on day 1 p.i., little inhibition of BBB damage was observed. Results were quantified to estimate the effects of Tempol in the BBB for whole brains (Figure 7B) and for olfactory bulbs (Figure 7C). Tempol thus seems to protect against BBB damage, but the effect is dependent on the starting day of administration.

### CM Survival in Mice Deficient in NOX2 (gp91^phox–/–^), SOD1, or in the Presence of SOD Inhibitor DETC or NADPH Oxidase Inhibitor DPI

**Figure 8:**
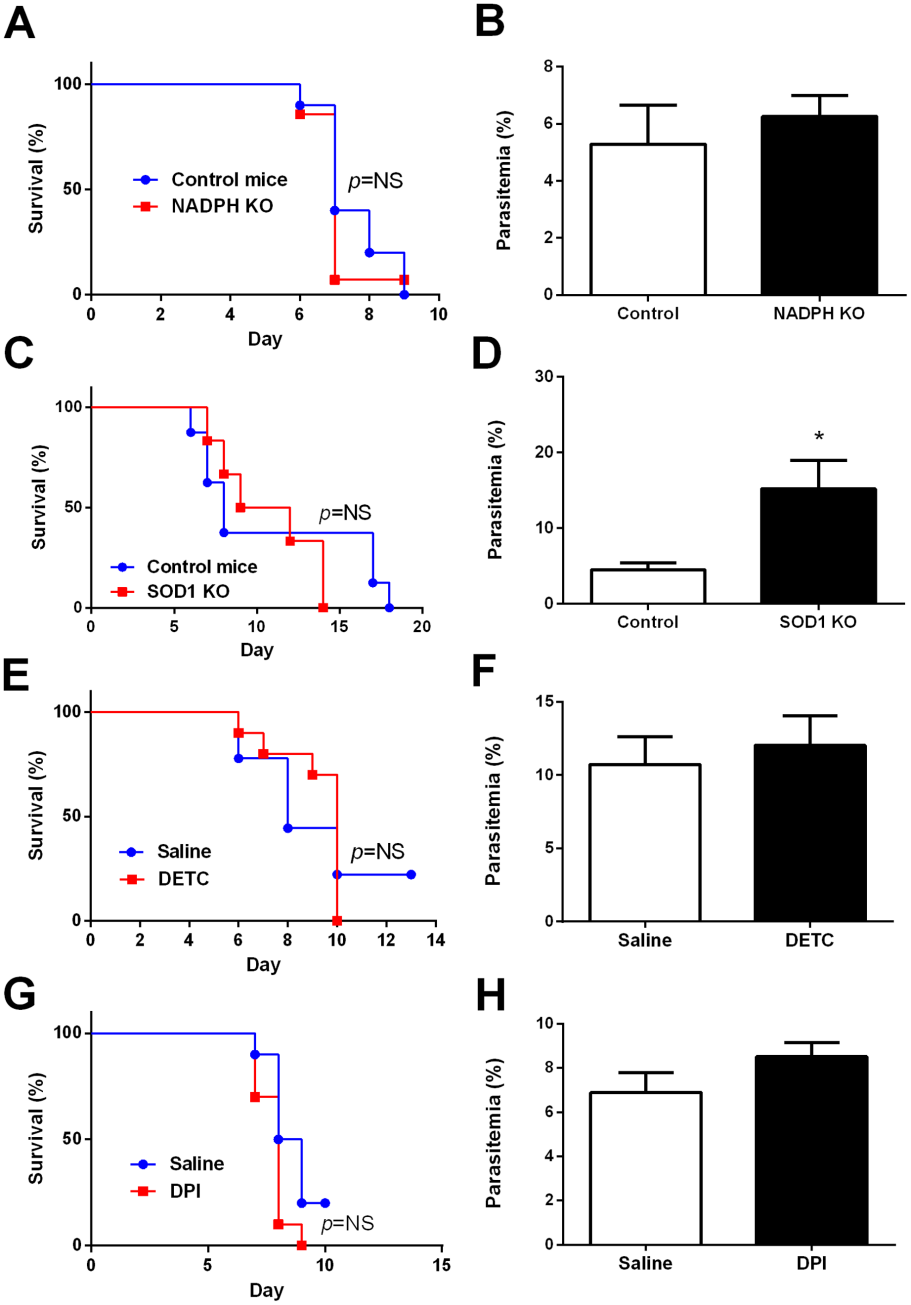
Cerebral malaria in mice deficient for NADPH oxidase or SOD1 or in the presence of DETC or DPI. *Plasmodium berghei* Anka parasitized red blood cells (10^6^) were injected intraperitoneally (i.p.) into **A,** mice knocked out (KO) for NADPH oxidase or controls (n = 8). **B,** An aliquot of blood was collected at day 6 to determine parasitemia. **C,** *P. berghei* Anka infection of mice KO for SOD1 (n = 8). **D,** An aliquot of blood was collected at day 6 to determine parasitemia. **E,** *P. berghei* Anka infection of mice treated with SOD inhibitor, DETC (100 µL, 50 mg/Kg) (n = 10). **F,** An aliquot of blood was collected at day 6 to determine parasitemia. **G,** *P. berghei* Anka infection of mice treated with NADPH oxidase inhibitor, DPI (100 µl, 2 mg/Kg)(n = 10). **H,** An aliquot of blood was collected at day 6 to determine parasitemia. *, P≤0.05 (Log-rank test). NS, non-significant.

To further understand the role of ROS in CM pathogenesis, we tested the effects of *P. berghei* Anka infection in KO mice for NOX2 (gp91^phox–/–^), which exhibits lower production of ROS. Figure 8A demonstrates that the curves obtained with gp91^phox–/–^ and age-matched congenic controls are essentially the same, which suggests that NOX2 alone plays a minor role on O_2_^−^-mediated vascular inflammation in this model. In addition, no change in parasitemia was observed for gp91^phox–/–^ infected mice (Figure 8B). We also tested the effects of infection in SOD1 KO mice, which exhibits impaired O_2_^−^ metabolism. Figure 8C shows that survival of SOD1 KO mice and controls are essentially the same, but parasitemia significantly increased (Figure 8D).We tested the effects of DETC, which inhibits SOD [86] and those of NADPH oxidase inhibitor DPI [87]–[90]. Our data show that DETC does not affect survival (Figure 8E) or parasitemia (Figure 8F). Likewise, the survival curves (Figure 8G) and parasitemia (Figure 8H) in the presence of DPI were essentially the same as controls.

### Sources of ROS in Malaria

**Figure 9:**
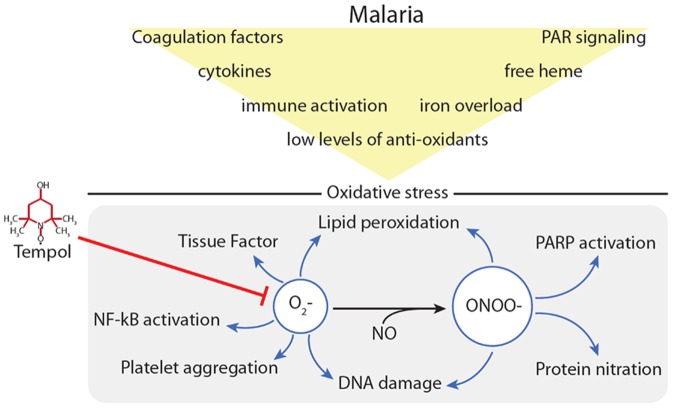
Pathologic events in malaria lead to radical oxygen species (ROS) generation and inflammation. *Plasmodium falciparum* infection is associated with coagulation and complement activation, cytokine release, host response to infection, and activation of different cells types, including endothelial cells, platelets, dendritic cells and monocytes, and neutrophils. It is also associated with release of *Pf*-GPI, iron overload, heme release and Fenton reaction, and diminished levels of antioxidants [1]–[9]. Superoxide contributes to platelet aggregation, tissue factor expression, cytokine release, NF-κB activation, DNA damage, NET formation, recruitment of inflammatory cells, conversion of nitric oxide (NO) to peroxynitrate (ONOO^−^) which in turn leads to DNA damage, PARP activation, lipid peroxidation, and protein nitration. These events result in sustained inflammation [52]–[56]. NO, nitric oxide; O_2_^−^, superoxide; ONOO^−^, peroxynitrate.

Figure 9 displays several mechanism that reportedly increase the pro-oxidant tonus in malaria, and the potential effects of Tempol.

## Discussion

*P. falciparum* infection is associated with increased levels of plasma cytokines, activation of the coagulation cascade, and signs of intravascular inflammation [1]–[9]. Inflammatory events are also associated with increased oxidative stress, and a pro-oxidant state has been reported in malaria [36]. Among ROS, O_2_^−^ is particularly important because it is the limiting step in generation of other free radicals (e.g. hydroxyl and peroxyl) [52], [53]. Superoxide is produced by NOX in the plasma membrane and cytoplasm, and by the electron transport chain in the mitochondria. Both origins of ROS participate in inflammation, and O_2_^−^ plays a critical role as a signaling molecule by activating redox-sensitive kinases [52], [53]. The assumption that ROS contribute to malarial pathogenesis has been the basis for studies devoted to understanding how antioxidants modify *P. falciparum* infection, experimentally or therapeutically [47]. In most cases, antioxidants such as NAC (in the absence of antimalarial agents) were not found to be effective [47], [59], [64]. While the effects of NAC are most commonly attributed to its capability to elevate cellular glutathione levels, it has limited cell membrane permeability properties and reacts slowly with O_2_^−^, H_2_O_2_, and peroxynitrate [65]. Conceivably, intracellular ROS remains relatively inaccessible to the direct or indirect effects of NAC when compared with antioxidants that strongly scavenge O_2_^−^ intracellularly [65].

Therefore a better understanding of how ROS—particularly intracellular O_2_^−^—contributes to the host response to *P. falciparum* infection is required. To this aim, we have evaluated whether the antioxidant Tempol [57] a SOD mimetic [98] that targets the intracellular compartment, affects cells involved in malaria pathogenesis in the context of coagulation and inflammation. LPS was used in vitro as a surrogate for *Pf*-GPI, a *P. falciparum*-derived molecule that is pro-inflammatory and pro-coagulant [26], through binding to TLR2/4 [39]. Various assays demonstrate that Tempol inhibits TF expression by ECs and that inhibition occurs upstream of transcription for TF as verified by real-time PCR. These results are congruent with the finding that Tempol prevents activation of NF-κB [99], which mediates transcription of the TF gene along with AP-1 and Egr-1 [92]. Tempol therefore resembles the effects of succinobucol (AGI-1067), an antioxidant that blocks TF expression in ECs and monocytes at the transcriptional levels [91]. Other antioxidants inhibit TF expression. For instance, resveratrol found in red wine reportedly inhibits transcription of TF [100]. Statins (e.g. rosuvastatin) prevent O_2_^−^ production by inhibiting isoprenylation of p21 Rac (which is critical for assembly of NOX) [101] and inhibit NF-κB activation and signaling pathways in ECs [67], [102]. Furthermore, simvastatin decreases monocyte TF expression and procoagulant/inflammatory state in mice, monkeys, and humans given a hypercholesterolemic diet [103] or LPS-induced monocyte TF expression in humans [104]. In experimental malaria, statins together with antimalarial agents decrease neuroinflammation and cognitive impairment in mice infected with *P. berghei* Anka by decreasing microvascular dysfunction, supporting the notion that ROS plays a role in disease pathogenesis [64]. It is conceivable that Tempol shares, at least in part, a similar mechanism of action with statins. Accordingly, Tempol's interference with the ROS/NF-κB/TF/PAR axis [3], [39] emerges as an additional, previously unrecognized potential anti-inflammatory property for the drug.

Our results also demonstrate that the effects of Tempol in EC in vitro were accompanied by decreased production of IL-6 (inflammatory cytokine), IL-8 (neutrophil chemoattractant), and MCP-1 (monocyte chemoattractant). In the context of *P. falciparum* infection, Tempol's effects in ECs are consistent with the protective effects of the antioxidant MnTBAP [105] and of intracellular SOD supplementation to ECs co-incubated with pRBC [106]. Because addition of *P. falciparum*-infected plasma to EC [107] or cytoadherance is associated with TF expression [10], ROS production [105], apoptosis [108], and activation of NF-κB [109], it is plausible that therapeutic targeting of O_2_^−^ would be effective to prevent the endothelial activation observed in severe malaria [110]. Furthermore, participation of NF-κB in malaria has been documented in mononuclear cells [111], macrophages [112], brain ECs [113], and in the liver [43], suggesting that the pleiotropic effects of Tempol could have potential therapeutic value.

DCs are professional antigen-presenting cells, and during a malarial infection also have a potentially direct pro-inflammatory effect through the release of cytokines upon interaction with pRBC [114], *Pf-*GPI [26], and coagulation factors [115], [116]. More recently, it has been found that DCs modulate host response to an infection by providing sustained coagulation activation through PAR1 and Sphingosine 1 phosphate pathways, which are critical to sustain decompensated coagulation responses [38] that may be present in CM [4], [11]. Because DC functions such as cytokine production, antigen presentation, and T cell stimulation are modulated by ROS [97], it was of interest to determine the effects of Tempol in DCs. Our results show for the first time that Tempol is an effective inhibitor of DCs functions, resulting in inhibition of TNF-α, IL-6, and IL12p70 production, impaired expression of co-stimulatory molecules and MHC class II, and CD4^+^ T cell stimulation. It remains to be determined whether this effect contributes to negatively modulate the function of T cells [117] in our model. Tempol also prevented the oxidative burst in neutrophil-like HL60 cells, which is known to promote endothelium activation through release of O_2_^−^ [118]. Likewise, Tempol attenuated platelet aggregation by collagen, which is partially ROS mediated [95] and associated with inflammation in malaria. Therefore, the effects of Tempol are better explained by a combination of effects on different cell types, all of which result in attenuation of local and systemic inflammatory tonus [57].

In vivo administration of Tempol (20 mg/Kg) started at day 1 p.i. was accompanied by a significant increase in survival. While Tempol-treated animals who survived were not completely healthy, they were consistently more active and responsive than untreated mice, as indicated by a trend for lower clinical score at day 6. Increasing the concentration of Tempol did not improve survival; paradoxically, the survival curves for mice given 60 mg/Kg (starting at day 1) were similar to saline-treated infected animals. These results suggest that a tight balance of the redox status [119]—which might have been dramatically altered by treatment with high doses of Tempol—is necessary for a physiologic host response to hypoxia and other changes associated with CM in mice. In other words, while an exacerbated production of ROS is detrimental, ROS is also required for normal homeostasis [120], and this balance might have been shifted by Tempol. Our in vivo results also demonstrated that the increase in survival after administration of Tempol is accompanied by lower plasma levels of MCP-1, an inflammatory cytokine produced by ECs that attracts monocytes and is a marker of vascular inflammation [121], [122]. A trend for lower levels of other inflammatory cytokines (e.g. IFN-γ and IL-6) was also found, corroborating the view that Tempol might attenuate the function of pro-inflammatory cells that play a role in CM pathogenesis [1]–[9]. Therefore, our interpretation is—based on the effects of Tempol in MCP-1 plasma levels and inhibition of platelets, neutrophils, ECs, and DCs in vitro—that the increase in survival promoted by the drug might be explained by its antioxidant actions in different cells involved in vascular inflammation. This effect might protect organs (e.g. liver, lung, and kidney) whose dysfunction contributes to severity of malaria [123]–[126].

In terms of brain pathology, the BBB has a critical role in CNS homeostasis, and intercellular tight-junction protein complexes of the brain microvasculature limit paracellular diffusion of substances from the blood into the brain [127], [128]. Hypoxia and reoxygenation, which are potential players of brain pathology in CM, are associated with production of ROS, which may lead to oxidative stress and dysregulation of BBB function [129]. Tempol appeared to be an ideal drug to be tested for its effects on BBB permeability, as it has been previously tested in hypoxia and reoxygenation in an in situ brain perfusion model and found to inhibit ROS-mediated increase in BBB permeability [130]. Tempol also preserves the BBB in murine viral encephalomyelitis [131]. In our experiments, Tempol given at day 4 p.i. and onward diminished the extravasation of IgG from the intravascular compartment to the parenchyma, indicating that it attenuated the increase in BBB permeability associated with CM; however, this effect alone is clearly not enough to increase survival, as most animals died similarly to infected, non-treated mice. Surprisingly, Tempol started on day 1 p.i. did not ameliorate brain inflammation at the BBB. This result suggests that the protective effects of Tempol started at day 1 p.i., which translate in increased survival, are not necessarily associated with blockade of ROS production specifically by the BBB, and possibly in the brain. In this respect, the role for ROS in brain pathology of malaria has been debatable. For example, it has been shown that increase in markers of oxidative stress in the brain occurs with non-CM forms (e.g. *P. berghei* K173), suggesting that global ROS is not specifically increased in experimental CM or is a good marker of disease severity in *Plasmodium* sp. infection [61]. More recently, an interesting study by Linares et al. [62] did not show an increase in ROS or nitrogen oxygen species in the brain of infected mice. It was also demonstrated that mRNA expression levels of SOD 1-3 and catalase in distinct regions of the brain are downmodulated during *P. berghei* Anka infection, as well as the expression of transcriptional factors controlling antioxidant host defenses. Also, no change in protein carbonylation was detected. Notably, the levels of heme-oxygenase and glutathione peroxidase are increased, suggesting that they compensate for the lack of SOD and catalase, counteracting oxidative damage and maintaining redox equilibrium [62]. In the case of Tempol started day 1 and onward, it is possible that impairment of antioxidant mechanisms may undermine the effects of Tempol, as SOD mimetics may also generate H_2_O_2_, which is proinflammatory, particularly under circumstances where low catalase activity is found such as in malaria, where increased SOD activity has also been reported. This may result in accumulation of H_2_O_2_, release of hydroxyl radicals (e.g. at the BBB), increased tissue damage during *Plasmodium* sp. infection, and lower efficacy for Tempol [49], [57], [132], [133].

Other antioxidants were tested in the ECM model. While PBN, a spin trap with ROS scavenging properties [79], did not protect against ECM significantly, it showed a trend for partial protection. Surprisingly, MnTE-2-PyP [78]—a well-known manganese-based porphyrin SOD mimetic that blocks inflammation in ischemia-reperfusion models [58]—was ineffective. On the contrary, it was toxic for infected mice and produced neurologic signs immediately after injections. It is possible that alteration of the BBB by *P. berghei* Anka infection might have contributed to increase toxicity of the MnTE-2-PyP, introducing a confounding that does not allows us to evaluate its protective effects unambiguously. MnTBAP, another porphyrin-based antioxidant that scavenges peroxynitrate in addition to O_2_^−^ [85], was ineffective in our model. Of note, survival curves in the presence of Mitotempo [134] or MitoQ [84], two antioxidants that accumulate in the mitochondria, were similar to those of infected, untreated animals. M30, a Fe^2+^ chelator and anti-antioxidant with neuroprotective effects in various neurodegenerative models of ALS, Alzheimer's, and Parkinson's diseases [81], was also ineffective. Finally, EGCG, the major green tea polyphenol antioxidant [80], did not change survival curves in our studies. It is plausible that the antioxidant activity of Tempol that targets O_2_^−^, H_2_O_2_, and peroxynitrate [57] explains why it is protective and the other antioxidants are not.

We also determined the effects of ROS in malaria pathogenesis by studying the effects of NOX2 (gp91^phox–/–^) deficient mice, which exhibit impaired production of O_2_^−^. Results presented in Figure 8 demonstrate that NOX2 KO mice survive similarly to the control group. Likewise, mice treated with NOX2 oxidase inhibitor DPI did not exhibit improved survival. These results are not surprising, as NOX2 is predominantly expressed in phagocytes, and much redundancy exists in regulation of ROS by other types of NOX, in their expression levels, and on their organelle localization [52], [53]. Likewise, survival curves were similar for SOD1 KO mice or for mice treated with the chemical inhibitor of SOD, DETC, which display impaired removal of intracellular O_2_^−^. While these results suggest that accumulation of O_2_^−^ or its products (e.g. H_2_O_2_) does not play a role in CM pathogenesis, interpretation demands caution, because there are multiple mechanisms for ROS scavenging (e.g. SOD1, SOD2, SOD3, glutathione peroxidase, catalase, heme-oxygenase) in vivo that might have compensated for the lack of SOD1, as recently reported [62]. In this context, ROS play a role in sepsis pathogenesis, but double KOs for SOD1 and glutathione peroxidase exhibit similar survival curves as control in the murine sepsis model [135]. Furthermore, mice overexpressing SOD1 and infected with *P. berghei* Anka develop oxidative injury associated with increased vulnerability to *P. berghei*, not protection, due to accumulation of H_2_O_2_ and Fenton reaction [136]. Conversely, the increase in parasitemia observed for SOD1 KO mice might have been due to impairment of formation of H_2_O_2_ or other ROS that might affect parasite survival in vivo.

Redox metabolism in *P. falciparum* is under control of several enzymes and cofactors that contribute to reducing the highly pro-oxidant tonus found inside pRBCs [137]. Notably, all antioxidants tested here, with the exception of PBN, promoted inhibition of *P. falciparum* growth in vitro with relatively low IC_50_ by mechanisms that remain to be determined [138]. Nevertheless, MnTE-2-PyP—which inhibited parasite growth with a remarkable low IC_50_ (2 µM) —was the only molecule to inhibit parasitemia in vivo (not shown), suggesting that it could be useful as a prototype to develop antimalarial agents once it is modified to avoid toxic effects.

Altogether, our results with Tempol in vitro and in vivo suggest that intracellular O_2_^−^ participates in malaria pathogenesis; however, no evidence that Tempol's protective effect (started day 1 p.i.) was mediated because it decreases inflammation in the BBB was found, based on IgG extravasation. Our results with Mitotempo and MitoQ likewise do not provide evidence to conclude that mitochondrial ROS is critical in CM pathogenesis. Of note, Karlsson et al. reported no differences in respiratory parameters of brain mitochondria between infected and non-infected mice, and no connection between disease severity and mitochondrial respiratory function was found in mice infected with *P. berghei* Anka [139]. Because we did not determine the ROS levels in different tissues and organelles, however, we cannot formally exclude a role for mitochondrial ROS in malaria pathogenesis. Conceivably, ROS production in malarial infection is regulated by the crosstalk and expression levels of several pro- and anti-oxidant enzymes and compensatory mechanisms in the brain [62] and possibly other organs. Finally, our results also need to be contextualized, given the differences observed between the infection caused by *P. falciparum* in mice and in humans [140].

Tempol is the first intracellular antioxidant found to partially increase survival in a CM model in mice. In the context of malaria treatment, systemic administration of Tempol in humans has not been evaluated, but topical use has been clinically tested and found to be effective against radiation-induced alopecia [66]. Modulation of ROS through therapeutic intervention in cardiovascular disease has also been attempted with antioxidants. Succinobucol—a well-studied antioxidant that prevents TF expression in monocytic cell lines [91]—has been tested in more than 6,000 patients with recent acute coronary syndromes and did not confer protective effects to justify its use therapeutically [141]. Likewise, treatment of Parkinson's disease in humans with MitoQ [142] was not effective despite promising experimental results [81]. Therefore, while intracellular ROS emerges as a novel potential therapeutic target for treatment of severe *P. falciparum* infection, the use of each antioxidant should be critically evaluated.
